# Psychological Experiences of Ocular Trauma and Traumatic Dental Injury Victims of Police Violence

**DOI:** 10.3390/dj13100481

**Published:** 2025-10-20

**Authors:** Gonzalo Rojas-Alcayaga, Andrea Herrera, Camila Corral Nuñez, Joaquín Varas, Sebastián Córdova, Carolina Lineros, Matías Ríos-Erazo

**Affiliations:** 1 Institute for Research in Dental Science, Faculty of Dentistry, University of Chile, Santiago 8380544, Chile; gorojas@odontologia.uchile.cl (G.R.-A.); aherrera@odontologia.uchile.cl (A.H.); carolina.lineros@ug.uchile.cl (C.L.); 2 Dental and Maxillofacial Service, Clinical Hospital, Universidad de Chile, Santiago 8380456, Chile; 3 Department of Restorative Dentistry, Faculty of Dentistry, University of Chile, Santiago 8380544, Chile; camila.corral@odontologia.uchile.cl; 4 Department of Occupational Therapy and Occupational Science, Faculty of Medicine, University of Chile, Santiago 8380453, Chile; joaquinvaras@uchile.cl; 5 Department of Oral Rehabilitation, Faculty of Dentistry, University of Chile, Santiago 8380492, Chile; scordova@odontologia.uchile.cl

**Keywords:** tooth injuries, psychological trauma, violence, police officers, patient care team, qualitative research, wounds and injuries, health care

## Abstract

**Background/Objectives**: Ocular trauma (OT) and traumatic dental injuries (TDI) inflicted by police officers not only cause significant physical harm, but also psychological trauma. The clinical attention given by health care teams may induce revictimization or retraumatization phenomena, which affect the psychological status of the victim. The objective of this research is to bring to light the psychological experiences related to emergency care processes and rehabilitation of people affected by OT and TDI caused by police violence. **Methods**: Qualitative research was conducted based on in-depth interviews with eighteen people affected by OT or TDI during the social outbreak in Chile in 2019–2020. Data analysis was based on the principles of grounded theory. **Results**: Three main categories emerged: quality of interpersonal relationships with health care providers, expectations of care and treatment and psychological consequences. The findings show that retraumatization and revictimization arise from clinical care in the context of state violence. **Conclusions**: Revictimization and retraumatization are the most characteristic phenomena occurring in the health care of people affected by OT and TDI caused by police violence. The probability of their occurrence depends mainly on the interpersonal relationships established with the health care team and the management of patient expectations regarding health care.

## 1. Introduction

In October 2019, Chile experienced a major sociopolitical upheaval known as the “social outbreak” (estallido social), which led to massive social movements during which agents of state forces used crowd-control weapons [1], and a high number of individuals were reported injured by firearms [2,3], specifically sustaining OT or TDI. Both are considered medical emergencies due to the proximity of these injuries to vital structures and their impact on organs that are critical for bodily function and appearance [4].

Regarding OT, the reporting of 460 severe cases over a two-month period (October to December 2019) [2] was classified as an “epidemic of eye traumas” by the Chilean Society of Ophthalmology [5]. This number significantly surpassed the 154 cases of eye trauma reported internationally over a six-year period in the context of armed conflict [6]. This type of trauma, defined as an injury to the eyeball and its peripheral structures caused by blunt or penetrating mechanisms, can lead to varying degrees of visual damage, functional impairment, and permanent disability [6,7]. Previous reports in Chile showed that over a 10-month period, the leading causes of ocular trauma care were primarily domestic, work-related, or school accidents. Firearms or kinetic impact projectiles (KIPs) (unclear causative agent) did not exceed 188 cases, representing only 1% of the total number of ocular trauma care visits [8].

There is little evidence regarding the prevalence and characteristics of TDI, defined as injuries caused by violent impact to the teeth and/or other hard or soft tissues in and around the mouth [9], during periods of social unrest. Previous reports on TDI estimated that it affects one-fifth of the population [10], primarily affecting a single tooth and being most prevalent in individuals under 20 years old [11]. TDI associated with KIPs has not historically been recognized as a highly prevalent cause.

In this context, a report on 77 individuals treated for these injuries at the University of Chile Dental Clinic is particularly valuable [12]. This report describes cases of people with multiple affected teeth and maxillary bone fractures from KIPs and physical force (such as punches and kicks) by the police. These findings highlight the potential for this type of weapon to cause damage to the maxillofacial area, often requiring complex physical rehabilitation and psychological support [13].

It is important to recognize that while both OT and TDI are covered for medical and surgical treatment under Chile’s “Explicit Health Guarantees” (Garantías Explícitas en Salud) state program [14,15], the psychological consequences of these traumas—especially when caused by KIPs and interpersonal violence from state agents—are not. The literature acknowledges that both OT and TDI can cause physical, psychological, emotional, and social consequences due to body image distortion and a temporary or permanent loss of autonomy, which leads to significant stress and difficulties.

Individuals who experienced facial disfigurement have been documented to face greater psychological challenges in adapting to their new condition, such as developing post-traumatic stress disorder (PTSD), compared to those with congenital disfigurement [16].

The traumatic event can be re-experienced in different ways. Typically, the person has recurrent and involuntary intrusive memories of the event, along with sensory, emotional, or physiological components, during which the event is re-experienced, and the person behaves as if it were happening in that moment. When exposed to triggers—events that are similar to or symbolize aspects of the traumatic event—an intense psychological or physiological response occurs [17].

Reeves (2015) defines retraumatization as the somatization of trauma, where memories can be triggered by physical examinations or procedures that cause them to re-experience negative feelings and emotions [18].

Given the above, it is relevant to consider that trauma experienced at the hands of state agents can also be defined in the literature as extreme trauma. This is because the person perceives a life-threatening situation from an authority figure with power, resulting in a profoundly disorganizing experience for cognitive functioning [19,20,21].

Studies on victims of state violence indicate that the development of PTSD and negative experiences in health care can lead to retraumatization. Victims have reported experiences of being unseen, silenced, and distrustful of care teams, as well as receiving negative attitudes from health care providers, poor quality of care, and experiencing disempowerment and a lack of decision-making ability. These experiences can be associated with retraumatization, whereas satisfaction and gratitude are recognized as protective resources [22].

Considering this scenario of extreme trauma and retraumatization associated with health care, guiding frameworks such as Dallam’s model of health care retraumatization are highly relevant. This model defines retraumatization as a cyclical process with four subprocesses: hypersensitivity to threats to safety, exposure to both sensory and relational triggers, post-traumatic stress reactions, and avoidant coping. The model proposes that when a patient experiences retraumatization, they develop an avoidant attitude toward future health care due to a strong emotional reaction and destructive coping attempts [23,24].

A comprehensive understanding of the psychological experiences associated with these types of traumas is even more relevant when considering the socio-historical context of Chile and the Latin American and Caribbean region, which is marked by structural inequality and massive social protests. Recently reported protests in countries like Nicaragua, Panama, Ecuador, Haiti, Peru, Chile, Bolivia, Venezuela, and Colombia between 2018 and 2019 [25] all had high levels of repression, but there were few reported psychological consequences, which confirms the lack of knowledge about the psychological consequences generated by extreme trauma. This emphasizes the need to understand the psychological experiences related to the emergency and rehabilitation care of people affected by TOIs and TDIs from state violence. In this sense, the present study is original in documenting, for the first time, the psychological experiences of victims of ocular and traumatic dental injuries caused by police violence, which is an area that has been largely overlooked in previous research.

The objective of this research is to bring to light the psychological experiences associated with emergency care and rehabilitation processes of individuals who have suffered OT and TDIs as a result of police violence.

## 2. Materials and Methods

The design of this study was exploratory research with a qualitative methodology. Data collection was conducted through in-depth interviews using a semi-structured guide and through the exploration of experiences related to the psychological reaction to the OT or TDI caused by police violence. Grounded theory was used as the method of data analysis.

(I) Ethical aspects

This study was conducted according to the guidelines of the Declaration of Helsinki and was approved by the Scientific Ethics Committee of the Hospital Clínico at the Universidad de Chile on 15 December 2021 (registered in minutes No. 78/20). All participants signed an informed consent before the start of the research.

(II) Participants

This research included women and men over 18 years old, with an age range of 20 to 61 years, from different regions of Chile who suffered OT and/or TDI caused by violent acts carried out by police officers in Chile, which were duly documented by some human rights organizations, health services, non-governmental aid and rescue, and health care teams at the Universidad de Chile.

The sample consisted of 18 participants who were recruited through theoretical sampling [26]; that is, data collection concluded when information saturation was reached.

Theoretical saturation was considered to have been reached when participants no longer contributed new experiences beyond those already identified in previous interviews via open coding. For instance, in interview number 18, no new experiences related to “quality of the interpersonal relationship” emerged that had not been mentioned in earlier interviews. To track the emergence of new experiences, an Excel spreadsheet was maintained with all emerging codes organized according to thematic axes. During the open coding of each interview, the spreadsheet was consulted to verify the emergence of any new code.

(III) Procedure

First, an interview was held with a psychologist from the Comprehensive Eye Repair Program (PIRO, according to its name in Spanish) as a key informant. This interview was transcribed and analyzed by three researchers to develop a script of questions for the following interviews. This interview script was piloted by each researcher individually with fifth-year dental students to construct the final version.

The recruitment of volunteers was conducted at the Dentomaxillar Prosthesis Clinic and Pediatric and Adult Dentoalveolar Traumatology Clinic in the Faculty of Dentistry, Universidad de Chile, as well as at the Low Vision and Visual Rehabilitation Unit of the Clinical Hospital of Universidad de Chile.

The researcher responsible for contacting the participant explained what the research was about and obtained informed consent. Then, an in-depth interview, which lasted approximately two hours, was conducted remotely due to the COVID-19 pandemic and restricted self-care measures. All interviews were recorded through Zoom and then transcribed verbatim for analysis.

Although memory bias can occur in any study where a substantial period of time has elapsed (2019–2020), in this case, given that the participants were survivors of physical and psychological trauma, we assumed that their recollection remained vivid. This assumption was supported by the interview process, as participants demonstrated high emotional engagement when discussing the events they had experienced.

(IV) Measures

The interviews were conducted by the research team comprising three academic psychologists from the Faculty of Dentistry and one academic occupational therapist from the Faculty of Medicine at the Universidad de Chile, all with experience in qualitative research methodology.

The interview script was organized into three parts: (1) introductory questions to build a trusting environment during the interview; (2) questions aimed at capturing immediate reactions to and consequences of the traumatic event, social environment, health care attention, and responsibilities for what happened; and (3) a closing statement as a summary that included suggestions for future interviews. The same interview guide, consisting of open-ended questions, was used for all interviews. The guide was progressively adjusted to accommodate emerging themes arising from the narratives of successive interviews.

(V) Data Analysis Plan

The analytical procedure consisted of coding the interview transcripts, where information was compared and common labels were assigned to diverse text segments that reflected the same underlying idea or concept (code). The identification and refinement of the codes were conducted collaboratively through a researcher–participant co-analysis process, ensuring that the coding framework emerged in dialog with the participants with respect to their lived experiences.

The analysis of the data gathered from the eighteen interviews consisted of three coding phases: open, axial, and selective. In the process of open coding, the software Atlas.ti^®^ Version 22.1.0 (ATLAS.ti Scientific Software Development GmbH, Berlin, Germany) was used. Relevant units of meaning were identified from the textual quotes obtained during the interviews, in accordance with the research objectives, and assigned a label or open code according to the following steps:

- Quotes:
  1. They didn’t judge me at all. In fact, they realized I was injured and tried to help me heal.
  2. They also provided me with immediate psychological support.
- Open code: Support from health care staff.
- Category: Appropriate relational models

Open codes were continuously compared and discussed among the team members. Then, axial coding was performed, with the objective of organizing the open codes into categories around an axis that integrated them with respect to their characteristics and scopes, allowing for the association of content and structure [27,28].

Finally, selective coding was performed to integrate all axial or emerging categories into a conceptual scheme, thus building an explanatory model [27,28].

During each coding phase, a triangulation process was conducted to achieve consensus among the researchers and specify the names of the codes, categories, and subcategories.

(VI) Quality criteria (rigor)

The criteria to ensure the rigor of this research were based on those proposed by Lincoln and Guba (1985) [29] to guarantee the quality of qualitative research. These criteria are credibility, transferability, dependability, and confirmability, as described below:

- Credibility: This criterion seeks to ensure that the interpretation of the information gathered corresponds accurately to the phenomenon under study and is not a bias of the researcher. To this end, it was ensured that the interviews were conducted at an appropriate time to allow the interviewees to express themselves as freely as possible. The propriety of the open coding and the definitions of the categories in axial and selective coding were executed through parallel programming and triangulation by four researchers from the project (GR-A, AH, JV, and MR-E).
- Transferability: This criterion refers to the applicability of the results to other populations in different contexts. This research was about a specific context in Chile. However, the methodology used regarding sample recruitment and information production allows for the results to be transferable to other contexts with similar conditions.
- Dependability: This criterion refers to the degree of consistency, reliability, and stability of the findings and interpretations throughout the research process. This is achieved by establishing a clear and well-documented research design that includes detailed descriptions of the purpose of the research, methods, and procedures of data collection. Moreover, it is achieved using rigorous and systematic techniques for data collection and analysis [30].
- Confirmability: The objective is to capture real-world phenomena without prejudice, allowing for the results to be confirmed in the data obtained from the interviews. In this study, after the final results had been obtained, triangulation was conducted with the interviewees to verify the categories and subcategories identified.
- Reflexivity: This criterion involves a process of self-awareness and reflective analysis by the researcher to continuously and critically evaluate the effect that a person has on the research and how it impacts interactions with volunteers. Such a reflective process is present at different levels of the study and phases, from formulating the research question to writing the final report [31]. In the present study, it is necessary to acknowledge the political stance of the researchers, given their supportive position regarding the demonstrations that occurred in Chile during 2019 and 2020, as well as their critical perspective on the actions of government authorities. This positionality undoubtedly influenced the interpretation of the participants’ statements and might have affected the identification of certain codes. However, throughout the research process, the researchers remained aware of this potential bias, taking it into consideration during both data analysis and their interactions with the participants.

## 3. Results

A description of the participants’ demographic characteristics, injuries, and the interval between sustaining the injury and the interview is presented in Table 1. Most participants were male (72.2%, *n* = 13). The mean age was 31.3 years (SD = 10.2). Of the 18 participants, 9 sustained OT, most frequently affecting the left eye. One participant presented with a penetrating orbital wound, and two participants had orbital fractures, both associated with ocular rupture; eyelid lacerations were reported in two cases. TDIs were observed in seven participants, involving hard dental tissues, pulp, and periodontal structures, with tooth involvement ranging from 1 to 5. The mean interval between injury and interview was 2.5 years (range: 1.6–3.8 years).

After analyzing the interviews regarding the health care experiences of these victims of OT and TDI caused by Chilean police officers, three main categories emerged: (1) quality of interpersonal relationship; (2) expectations about care and treatment; and (3) psychological consequences. A summary of the categories is presented in Table 2.

### 3.1. Quality of Interpersonal Relationships

This category gave rise to three subcategories: (1) appropriate relational models, (2) inappropriate relational models, and (3) structural conditions of the health care system.

#### 3.1.1. Appropriate Relational Models

The relational models of care correspond to the communicative interaction between the patient and the health care provider during the delivery of care. These relational models can be appropriate or inappropriate. In appropriate relational models, the interviewees described the health care they received as compassionate, empathetic, and friendly, characterized by humanized, personalized, and sincere care, which is reflected in the respectful and accurate way of providing sensitive information.

Both genuine interest in the patient and emotional support provided throughout the care process generate a strong bond between the health care provider and the patient, favoring physical and psychological improvements.

*“Why am I so grateful? Because they took time to get in touch with me, look for me, send me emails, and find out if I am able to attend an appointment.” (E.10)*

The interviewees appreciate that their health care providers could offer clear and comprehensible explanations, avoiding or reducing the use of technical terms.

*“How they explained things to me, how they treated me without making me feel bad or anything like that, they tried to address the topic as simply as possible, explaining everything so that I could be calm.” (E.12)*

#### 3.1.2. Inappropriate Relational Models

The inappropriate relational models were associated with depersonalized or dehumanized care, in which interviewees reported not receiving the expected emotional support from their health care providers despite the complexity of their situation, along with insufficient information and guidance provided to patients.

*“…the ophthalmologist was asking for information, filling out paperwork, and never looked at me. I asked him questions, and he never answered them, and he only cared about signing the paperwork.” (E.6)*

When health care providers fail to recognize patients as individuals who are suffering, minimize the role of communication in care, and restrict themselves to a purely technical role, patients often experience dissatisfaction. A lack of empathy, particularly during invasive procedures, reflects a form of dehumanization in the care of a person who has suffered psychological trauma.

*“I remember that the dentist told me… if you do not stop crying, the anaesthesia will not take effect, and I will have to pull it out (the tooth) like this (without anaesthesia).” (E.13)*

#### 3.1.3. Structural Conditions of the Health Care System

Structural conditions involve elements of human resources, infrastructure, and organizational aspects, which cannot be modified because they represent constitutive elements in all health care systems. However, they significantly influence patients’ perceptions of the quality of interpersonal relationships with the health care team. The main aspects that the interviewees complained about are the waiting time, boring administrative procedures, and the lack of integration in the health care system.

*“To schedule an appointment, there were a lot of procedures to do at the hospital; I had to leave my appointment in the mailbox and wait for them to call me.” (E.9)*

The absence of physical resources and specialized medical staff, mainly ophthalmologists and psychiatrists, is an important aspect that influence patients’ perceptions of the quality of the health care received.

*“They called the specialist to see the wound but at that time (Friday 8 pm), the specialist was not there and they did not have a maxillofacial in the emergency room either… so they told me that on Monday I had to go to the trauma hospital.” (E.2)*

*“It was a lot of trouble that they gave me sedatives because there were only nurses, and nurses can’t give sedatives” (E.1)*

The interviewees perceive an absence of integration in the Chilean health care system, which is described as fragmented and noticeably distinguishable between public and private health care.

*“I arrived at the hospital; I did not even get out (of the ambulance) and they said: no! she has private health insurance. We can treat you, but it is going to be expensive.” (E.6)*

### 3.2. Expectations of Care and Treatment

This category gave rise to three subcategories: expectations toward the rehabilitative treatment, active role of the patient, and expectations of psychological support.

#### 3.2.1. Expectations Towards the Rehabilitative Treatment

The interviewees reported greater satisfaction with their treatment when their treatment sessions were prompt. In this sense, the COVID-19 pandemic made the situation even more complex due to the suspension of elective health care appointments. The interviewees were affected as appointments in public health care had to be postponed.

*“…the time between sessions was prolonged and the pandemic, made the available hours even more scarce… I do not know how it is in the private system, but it was two years; I think if it had been shorter, it would have been better.” (E.5)*

On the other hand, when surgical procedures were carried out opportunistically, it resulted in satisfaction with the treatment.

*“I liked a lot the fact that I was operated on quickly (timely).” (E.6)*

The interviewees highlighted the importance of preferential and comprehensive programs for victims of violence perpetrated by police officers.

*“I would have liked to have neurological rehabilitation or maybe a physical therapist. I think the health care system has a lot of shortages, services that could be performed, or things that we need.” (E.6)*

The loss of teeth or eyesight has severe consequences for the self-image and self-esteem of affected individuals. For this reason, having prosthetic rehabilitation generates a sense of satisfaction in the patient.

*“When I started using the prosthesis, I was happy; the doctor showed me how it would look; step by step, each session made me happier because before the prosthesis, I had nothing; it was empty.” (E.11)*

#### 3.2.2. Active Role of the Patient

When patients have an active role in their treatment, it increases their satisfaction, whereas reducing their participation in the process may be an obstacle in the patient–doctor relationship.

*“It has been a good experience with the prosthesis, but this was a slip-up, feeling that I cannot give my opinion.” (E.6)*

Satisfaction with treatment is a feeling that emerges when health care is prioritized and provided promptly, when high-quality treatment is delivered, and the cosmetic results meet the patient’s expectations. Another key aspect related to the active role of patients is the collaboration between health care professionals and patients.

*“The main part of the treatment is already finished; I have my permanent teeth now. We are in the last cleaning and polishing session, but it is almost ready. And the support I have received here has been incredible.” (E.5)*

#### 3.2.3. Expectations of Psychological Support

The expectations of psychological support have two aspects: the availability of psychotherapy and effective psychological support. Regarding psychotherapy, it is a necessity to help patients deal with traumatic events.

*“I think I will always need psychological therapy, at least when I talk about my story, venting, and reflecting… trying to come to a conclusion that helps me to cope with it.” (E.6)*

Likewise, mental health support is expected to be effective; that is, it must lead to significant improvements in the general condition of patients affected by this type of trauma.

*“I remember that the first month I took another therapy offered by someone from the College of Physicians, and it made me feel in my right mind. I think that without it, I would not be able to speak so lucidly now.” (E.1)*

### 3.3. Psychological Consequences of Health Care

This category gave rise to two subcategories: retraumatization and revictimization.

The physical aggressions experienced by the interviewees produced functional limitations and esthetic and psychological impacts. However, not only the original physical trauma but also the procedures performed during treatment caused negative consequences, including in the form of psychological trauma. The main psychological reactions triggered by rehabilitative treatment are retraumatization and revictimization. Both phenomena exacerbate and perpetuate the initial psychological trauma.

#### 3.3.1. Retraumatization

In some cases, the therapeutic process could trigger a retraumatization effect in the affected person due to the use of surgical tools that produce feelings like those caused by the original traumatic stimulus.

*“Every time I heard the sound of the turbine, I knew that what they were going to do would hurt; it was like bringing me back to that moment (TDI).” (E.5)*

Certain clinical procedures make the patients re-experience the feelings of physical trauma, producing anxiety during the clinical session, as described in the following case involving an impression procedure of the eye socket for prosthetic elaboration.

*“I felt it in my body; I felt bad; I had panic attacks, and those kinds of things. I felt again things like the ringing or the pain in the face, and all of that. Thus, when they put that funnel (eye) and other similar things that are warm (interviewee refers to the impression material) … that feeling was triggered immediately. (E.1)*

Recurrent memories about traumatic events are frequent, occurring in dreams or nightmares, along with emotional distress or physical reactions linked to the trauma. These symptoms can be triggered by rehabilitative treatment.

*“Most of the rehabilitations made me relive my story and remember what had happened to me so that they could be aware of it and help me. Every time that I told my story, for me, that topic was cold… at that moment, but at night when I had the same dreams, I woke up crying, sad, and screaming.” (E.7)*

#### 3.3.2. Revictimization

Revictimization has a greater impact on satisfaction or dissatisfaction with the care received because it is directly related to the interpersonal relationship being perceived as an aggression on human rights and dignity. It occurs when the affected person has to tell the traumatic event repeatedly.

*“It was difficult because I had to recount the same story, because they had noticed that the anxiety was still there when I started the treatment. Thus, telling my story makes me sad, just like now.” (E.13)*

**Figure 1:**
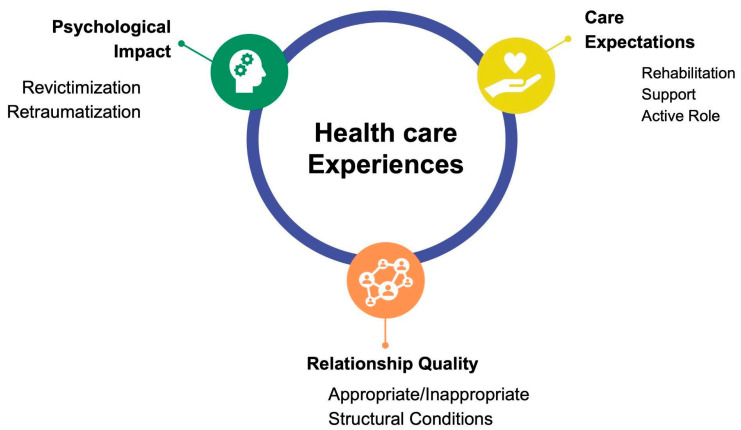
Relationship between the three emerging categories.

Although the experiences described above may occur in any type of emergency health care, the existence of a previous traumatic event worsens the psychological response. In this case, the origin of the traumatic event is violence perpetrated by police officers. The victims were exposed to intentional aggression, resulting in feelings of vulnerability. If the health care team does not consider these aspects, the victims may undergo experiences that deepen the initial psychological damage. Therefore, an appropriate relational model and the management of expectations of care can help to reduce undesirable consequences for the health and well-being of people affected by police violence. Figure 1 presents a diagram of the relationship between the three emerging categories, which are interrelated and describe the health care experiences of victims of OT and TDI caused by Chilean police officers.

## 4. Discussion

The findings of this research demonstrate that people who have suffered physical trauma caused by police violence present greater psychological vulnerability necessitating the attention of health care teams, both in terms of relational aspects and the clinical procedures applied. Moreover, the structural conditions of the health care system in which the relationships between patients and health care providers take place play a significant role and can exacerbate the difficulties of providing and receiving clinical care. This situation of vulnerability produces expectations regarding health care attention that are largely unmet, generating dissatisfaction. Retraumatization and revictimization are the main psychological consequences of health care when structural deficiencies of the system are not considered, especially given the importance of appropriate relational models and the management of patient expectations.

It is important to mention that, for the purpose of this research, a distinction was made between the phenomena of revictimization and retraumatization. In the literature, revictimization is understood as distress caused by state institutions on victims [32], and retraumatization is the reproduction of trauma by state terrorism [33]; therefore, the two terms are considered synonyms. However, for the purpose of this research, revictimization means “considering to be a victim again” (from the relational aspect), and retraumatization means re-experiencing the trauma, mainly referring to the sensory aspects caused by physical stimuli.

OT and TDI have significant psychological consequences because they are injuries to body parts that hold important representation for a person’s body image and identity. It has been described that OT, regardless of the cause, results in severe psychological consequences in the affected individual, such as depression and post-traumatic stress. Moreover, physical damage to the face and the mouth, mainly the teeth, is associated with severe psychological consequences, including the development of PTSD, especially in young people [34]. The quality of life related to oral health is also significantly affected in people with oral damage. The financial cost of treatment is another relevant aspect. However, unlike our study, previous research shows that OT or oral/facial trauma is mostly caused by work, traffic, or household accidents, rather than by violent actions of police officers. There are common denominators for these situations, but a completely equivalent comparison of the psychological consequences of OT and TDI is not possible. Nevertheless, we can assume that in the case of OT and TDI caused by the violence of police officers, the psychological consequences are even more serious.

It is important to note that the presence of PTSD symptoms following oral and facial trauma can be identified early, even at the moment of the emergency care [34]. Hence, the structural conditions of the health care system and the relational models in which health care takes place play an important role in the treatment of psychological consequences associated with ocular and maxillofacial trauma. The situation is even more critical when the physical trauma has been caused by police officers, as it is a situation of extreme trauma.

Patient perceptions of health care quality appear to be significantly shaped by prior traumatic experiences. From a phenomenological standpoint, however, the central issue is not the objective accuracy of the medical care received, but rather the way in which it is subjectively experienced and narrated by the traumatized individual. This distinction underscores the importance of acknowledging that patients’ perceptions of care may diverge from biomedical assessments of quality, while still exerting a profound impact on their trust in health care systems, adherence to treatment, and overall well-being. Consequently, understanding health care quality through the lens of lived experience is essential for developing more patient-centered approaches that address both clinical and existential dimensions of care.

In general terms, revictimization of any origin increases the probability of suffering PTSD, which is seen, for instance, in women who have suffered sexual abuse and then revictimization [35]. In this sense, the revictimization of people with OT or TDI undoubtedly increases the risk of PTSD, even though this association was not evaluated in this research.

The health care attention of people who have suffered any type of psychological trauma inflicted by police officers is a sensitive topic that requires adapting existing relational models and procedures to avoid inducing experiences of revictimization or retraumatization. In the case of people who have been victims of torture, it is suggested that health care teams be aware that they are treating people who are in a vulnerable psychological condition. For this reason, they cannot apply general care protocols and must adjust times, spaces, communication styles, and procedures in order to build trust and security for the person who receives clinical care [22]. In this regard, it must be considered that people who have suffered psychological trauma resulting from violent actions that endanger their physical and mental integrity have lower trust in other people, which may affect the therapeutic relationship with the health care team [36].

Appropriate relational models are characterized by compassionate and personalized care, effective communication, and emotional support. Conversely, a lack of empathy and patient invisibility were identified as inappropriate models. These undermine trust, risk making patients feel judged, and compromise the therapeutic alliance [37].

Medical teams’ responses to patient demands are frequently insufficient, as they often neglect the sociocultural and contextual factors underlying the health problem. Health care professionals tend to interpret clinical situations primarily through the normative frameworks and epistemological constructs of their discipline, rather than integrating patients’ values, priorities, lived experiences, and belief systems. This reductionist approach may compromise the therapeutic alliance, potentially leading to a critical deterioration in the patient–provider relationship, wherein patients perceive their specific health circumstances as unacknowledged or invalidated [38].

Our results highlight the central role of the therapeutic bond in health care [39,40,41]. In this regard, it is essential to develop guidelines that help prevent revictimization and appropriately manage clinical procedures that may pose a risk of retraumatization [14]. Implementing such measures is crucial for reducing long-term psychological consequences, including post-traumatic stress disorder. Although international protocols exist, they are mainly focused on cases of child abuse or domestic violence [42]. However, very little research has examined police-inflicted trauma within health care contexts. This study, therefore, contributes to a deeper understanding of its causes and provides evidence to guide the development of specific recommendations for health care teams.

The results regarding the influence of structural conditions on the quality of the interpersonal relationship between the health care team and the patient are consistent with findings related to patient satisfaction regarding the quality and conditions of health care [43]. Previous studies already reported a shortage of specialists and feelings of vulnerability as negative aspects, in addition to the perception that “essential changes are needed” in the public health system. Regarding health centers, the same research highlights that the manners of administrative workers and doctors “need to be improved.” These are aspects that are recognizable in the experiences explored in this research.

The Chilean health care system is structured as a mixed model comprising a public insurance scheme, known as FONASA, and a private insurance scheme, referred to as ISAPRE. Health care services may be delivered as part of both public and private facilities. Public institutions provide all types of care, generally upon referral from a primary health care center (Consultorio, CESFAM, or equivalent), except in cases of emergency care [44]. Primary emergency services (SAPU/SAR) are available to anyone requiring medical attention. One of the main challenges faced by these services is the prolonged waiting times to receive care. According to data from the Ministry of Health (MINSAL), in 2022, within the South-Eastern Metropolitan Health Service and Central Metropolitan Health Service, 30% and 37% of patients, respectively, waited more than 12 h for a hospital bed [45].

Taking the above into consideration and according to our results, the expectations of timely and appropriate treatment and availability of effective psychological support for the traumatic event, as well as the expectations of having an active role and participation in the care processes, are elements or principles that any treatment program should include, in recognition of the guidelines of national and international human rights organizations for the reparations of crimes and human rights violations. These guidelines establish that the reparation must be comprehensive, appropriate, fast, effective, and proportionate to the severity of the situation. Rehabilitation is one of the five fundamental principles for response mechanisms, alongside the principles of satisfaction, guarantees of non-repetition, restitution, and compensation—aspects that should be assembled within health care processes [46,47,48].

Finally, the experiences described in this research indicate the need to include new actions into clinical or technical guidelines for OT and TDI in Chile [7,14,15]. The current guidelines lack the recognition of a human rights perspective that includes preventive aspects of psychological trauma associated with health care. In this sense, the incorporation of these aspects is an essential and truly relevant element for addressing psychological and social trauma, together with the continuity of care and follow-up for affected individuals.

This study had some limitations. Although the principle of saturation was met, we acknowledge that the sample of volunteers is small and could have been expanded; however, recruitment was not easy, given the psychological implications of the trauma itself. Most of the interviews were conducted with patients from Santiago, Chile, which limits a more comprehensive understanding of the experiences, given the possible differences arising from place of residence. In addition, the interviews were conducted via Zoom due to the pandemic and restricted self-care measures, and this context might have either facilitated or hindered the participants’ expression during the interview. Finally, patients with other bodily injuries were not included in the sample, since we focused on facial structures that have a significant impact on body image and were those that generated the greatest social impact during the social outbreak.

Although psychological trauma is thought to preserve vivid memories of events, the certainty of this effect cannot be assumed due to the two-year interval between the traumatic event and the interview. This temporal gap, therefore, represents a methodological limitation in the interpretation of the study’s findings, since the narratives could have been distorted by memory bias.

Future research should address the medium- and long-term psychological consequences of OT and TDI in victims of police violence through longitudinal studies, while also incorporating comparative international perspectives to assess the transferability of findings across different contexts of state violence and civil conflict. Further efforts should also be directed toward the development and evaluation of trauma-informed and human rights-based clinical protocols specifically designed for this population, complemented by quantitative studies that measure the prevalence and severity of psychological symptoms. Interdisciplinary and transdisciplinary approaches will be essential to integrate health care, social, legal, and community dimensions, ultimately guiding the creation of comprehensive models of care and reparation that respond to both clinical and psychosocial needs.

## 5. Conclusions

This study highlights that revictimization and retraumatization are the most distinctive psychological consequences in the health care of individuals affected by OT and TDIs caused by police violence. Their occurrence largely depends on the quality of the therapeutic relationship and the management of patient expectations.

To address these challenges, health care systems should move beyond individual clinical recommendations and adopt a structural, trauma-informed and human rights-based approach. This implies the development of the following national clinical guidelines:

- Incorporate empathetic communication and procedural adaptations to minimize retraumatization;
- Ensure timely and continuous psychological support as an integral component of care;
- Provide specific training for health care teams (including legal and psychosocial dimensions) to equip professionals with the skills to manage extreme trauma and avoid revictimization.

From a public health and policy perspective, these findings support the creation of a permanent, state-led reparation and rehabilitation program for victims of institutional violence. Such a program should include the following:

- Explicitly recognize violence by state agents as an extreme traumatic experience requiring priority access to comprehensive, reparative health care;
- Secure sustained public funding for long-term rehabilitation, including prosthetic, dental-maxillofacial, and mental health care;
- Implement protocols and training plans for all levels of the public health network to prevent retraumatization, support victims during legal and administrative procedures, and ensure continuity of reparative care throughout the life course;
- Integrate an intersectoral perspective, connecting health services with social, legal, and occupational support to restore victims’ life projects and guarantee non-repetition.

By translating these clinical implications into trauma-informed policies and institutional guidelines, the health system can shift from fragmented and reactive responses toward a dignified, comprehensive, and reparative model of care aligned with international human rights standards.

## Figures and Tables

**Table 1:** Demographic and clinical characteristics of participants, including injury type and injury-to-interview interval.

Code	Gender	Age (Years)	Type of Injury	Injury Description (ICD-11)	Injury-to-Interview Interval (Years)
1	M	18	OT	NA06.8: Traumatic injury to eyeball (ocular rupture, left eye)	1.60
2	F	42	TDI	NA0D.0: Injury of hard dental tissues and pulp (2 teeth); NA0D.1: Injury of periodontal tissues (3 teeth)	1.71
3	M	24	TDI	NA0D.0: Injury of hard dental tissues and pulp (1 tooth); NA0D.1: Injury of periodontal tissues (1 tooth)	1.73
4	F	61	OT	NA06.1: Penetrating wound of orbit with or without foreign body (right eye)	1.86
5	M	24	TDI	NA0D.0: Injury of hard dental tissues and pulp (3 teeth); NA0D.1: Injury of periodontal tissues (3 teeth)	1.88
6	F	27	OT	NA06.8: Traumatic injury to eyeball (ocular rupture, left eye)	2.23
7	M	21	TDI	NA0D.0: Injury of hard dental tissues and pulp + NA0D.1: injury of periodontal tissues (2 teeth); NA0D.0 (5 teeth)	2.21
8	M	43	OT and TDI	NA0D.0: Injury of hard dental tissues and pulp (1 tooth); NA0D.1: Injury of periodontal tissues (2 teeth)	2.39
9	M	24	OT	NA06.8: Traumatic injury to eyeball (ocular rupture, left eye)	2.48
10	M	28	OT	NA06.8: Traumatic injury to eyeball (ocular rupture, right eye); NA02.2: Fracture of orbit (right eye)	2.51
11	M	30	OT	NA06.8: Traumatic injury to eyeball (ocular rupture, right eye); NA02.2: Fracture of orbit (right eye); NA06.0: Eyelid trauma (eyelid laceration, right eye)	2.65
12	M	26	OT	NA06.8: Traumatic injury to eyeball (ocular rupture, left eye)	2.80
13	F	32	TDI	NA0D.0: Injury of hard dental tissues and pulp + NA0D.1: Injury of periodontal tissues (1 tooth); NA0D.0 (2 teeth)	2.66
14	M	36	TDI	NA0D.1: Injury of periodontal tissues (3 teeth)	2.92
15	F	25	OT	NA06.8: Traumatic injury to eyeball (ocular rupture, left eye)	2.94
16	M	31	OT	NA06.8: Traumatic injury to eyeball (ocular rupture, left eye)	2.99
17	M	26	TDI	NA0D.0: Injury of hard dental tissues and pulp (1 tooth)	3.07
18	M	30	OT	NA06.8: Traumatic injury to eyeball (ocular rupture, left eye); NA06.0: Eyelid trauma (eyelid laceration, right eye)	3.84

OT: ocular trauma; TDI: traumatic dental injury.

**Table 2:** The main categories and their subcategories related to the health care experiences of victims of OT and TDI caused by Chilean police officers.

Main Categories	Subcategories		
Quality of interpersonal relationships	Appropriate relational models	Inappropriate relational models	Structural conditions of the health care system
Expectations about care and treatment	Expectations towards the rehabilitative treatment	Active role of the patient	Expectations of psychological support
Psychological consequences of health care	Retraumatization	Revictimization	

## Data Availability

The raw data supporting the conclusions of this article will be made available by the authors upon request.

## Abbreviations

The following abbreviations are used in this manuscript:

OT	Ocular trauma
TDI	Traumatic dental injuries
KIPs	Kinetic impact projectiles
PTSD	Post-traumatic stress disorder
